# Modulation of Immune Responses by Nutritional Ligands of Aryl Hydrocarbon Receptor

**DOI:** 10.3389/fimmu.2021.645168

**Published:** 2021-05-20

**Authors:** Alba De Juan, Elodie Segura

**Affiliations:** Institut Curie, PSL Research University, INSERM U932, Paris, France

**Keywords:** aryl hydrocarbon receptor, AhR, intestine, microbiota, tryptophan, immunity

## Abstract

Accumulating evidence indicates that nutrition can modulate the immune system through metabolites, either produced by host digestion or by microbiota metabolism. In this review, we focus on dietary metabolites that are agonists of the Aryl hydrocarbon Receptor (AhR). AhR is a ligand-activated transcription factor, initially characterized for its interaction with xenobiotic pollutants. Numerous studies have shown that AhR also recognizes indoles and tryptophan catabolites originating from dietary compounds and commensal bacteria. Here, we review recent work employing diet manipulation to address the impact of nutritional AhR agonists on immune responses, both locally in the intestine and at distant sites. In particular, we examine the physiological role of these metabolites in immune cell development and functions (including T lymphocytes, innate-like lymphoid cells, and mononuclear phagocytes) and their effect in inflammatory disorders.

## Introduction

Food represents not only a source of nutrients for the maintenance of essential biological functions, but also contains dietary components that regulate immune cell populations. These include microbiota-derived short-chain fatty acids, polyamines, and indoles derivatives, which are ligands of the Aryl Hydrocarbon Receptor (AhR) (1).

AhR is a ligand-activated transcription factor residing in the cytosol. Upon binding to an agonist, AhR translocates to the nucleus where it forms an active heterodimer with ARNT and promotes the transcription of genes that are under its control. AhR is expressed in multiple immune cells such as myeloid cells, innate lymphoid cells, B lymphocytes and certain subtypes of T cells. AhR activation has an overall anti-inflammatory and immunoregulatory role in innate and adaptative immunity, both in steady-state or in inflammatory scenarios such as autoimmunity or infection (2). However, a number of these observations were made *in vitro* or *in vivo* by injecting AhR agonists in non-physiological routes or concentrations. Some studies have also used AhR ligands of xenobiotic origin, which are known to induce aberrant AhR signaling (3). This has led to some contradictory findings, in particular in T cell biology (4).

In this review, we examine the physiological role of nutritional AhR ligands in immune cells and immune responses by focusing on experimental results obtained by direct intestinal exposure or diet manipulation.

## What Are the Nutritional AhR Ligands?

**Figure 1:**
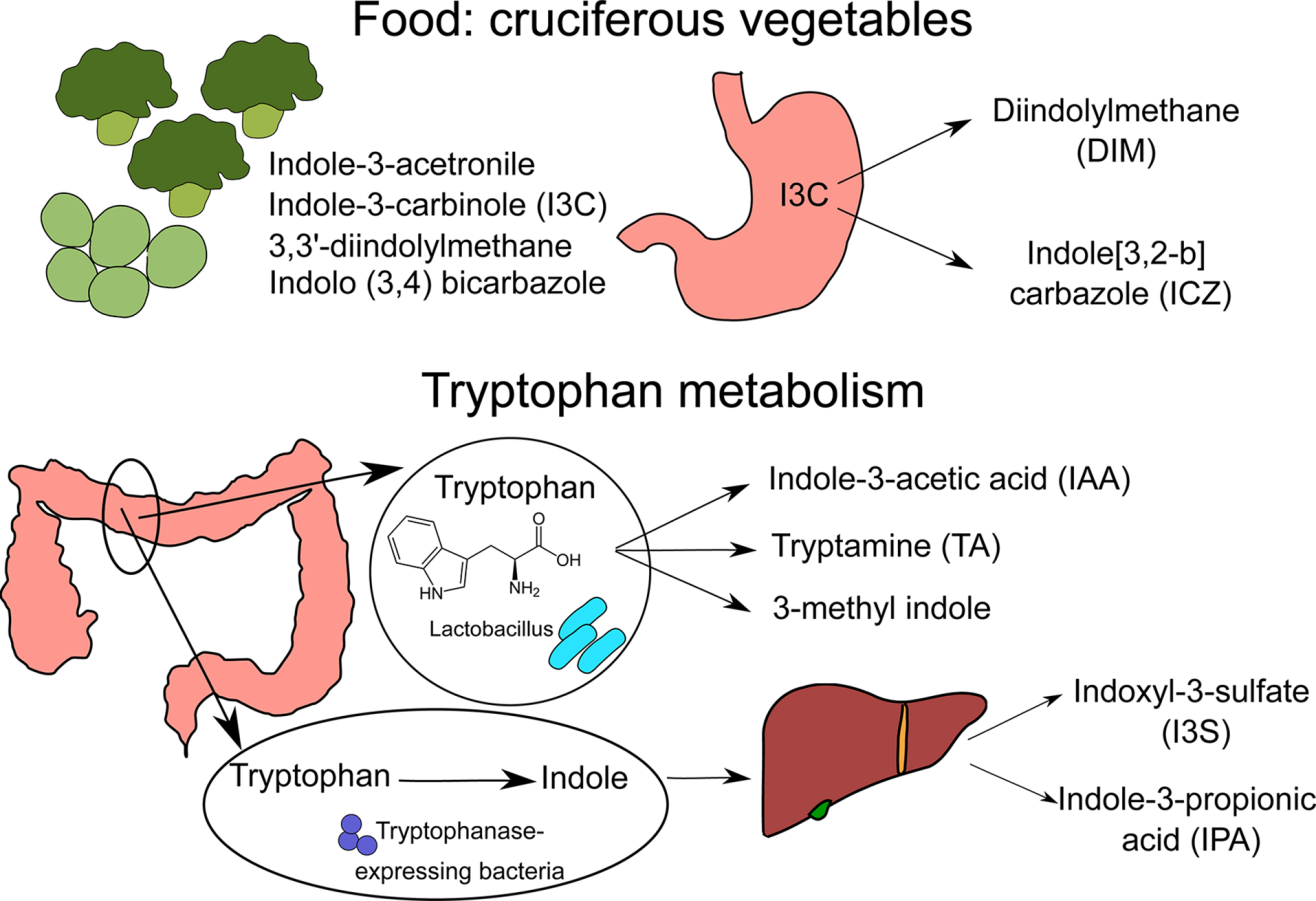
Sources of nutritional AhR ligands. Nutritional AhR ligands are derived either from the breakdown of food components or from tryptophan catabolism by intestinal microbiota. Several types of indoles are present in cruciferous vegetables. In particular, indole-3-carbinol (I3C) is converted in the stomach into high affinity AhR ligands Diindolylmethane (DIM) and indole[3,2-b]carbazole (ICZ). Tryptophan is metabolized by *Lactobacillus* bacteria into indole-3-acetic acid (IAA), tryptamine (TA) and 3-methyl indole. In addition, tryptophanase-expressing bacteria degrade tryptophan into indole, which is metabolized by host liver cells into indoxyl-3-sulfate (I3S) and indole-3-proprionic acid (IPA).

AhR was initially described as a receptor for xenobiotic pollutants, mostly aromatic hydrocarbons. However, over the years, physiological ligands have been identified. There are other exhaustive reviews of AhR agonists (5–7), here we focus on nutritional AhR ligands (**Figure 1** and **Table 1**).

**Table 1:** Nutritional AhR ligands classification.

Compound	Abbrevations	Source	Reference
Quercertin		Dietary ligands	(8–10)
Resveratrol			
Indole-3-acetonitrile	IAN		(11)
Indolo(3,4)bicarbazole			
Indole-3-carbinole	I3C		
3,3'-diindolylmethane	DIM	Host metabolism from food components	
Indole[3,2-b] carbazole			
Linear trimer, [2-(indol-3-ylmethyl)-indol-3-yl]indol-3-ylmethane	LTR_1_		(12)
1-(3-hydroxymethyl)-indolyl-3-indolylmethane	HI-IM		
5-hydroxyindole-3-acetic acid	5-HIAA		(13)
Indole-3-acetic acid	IAA	Microbiota metabolism from food components	(14, 15)
Tryptamine	TA		
3-methyl indole			
Indole-3-aldehyde	IAld		(15)
Butyrate			(12)
Indoxyl-3-sulfate	I3S	Host metabolism from tryptophan catabolites from microbiota	(16)
Indole-3-propionic acid	IPA		

Reported nutritional AhR agonists classified based on their origin.

AhR ligands are present in the diet, as natural compounds in food. Flavonoids derived from fruits and vegetables such as quercertin and resveratrol have been identified as AhR ligands based on *in vitro* assays, but their physiological relevance is unclear (8–10). The main class of dietary AhR ligands is indoles, including Indole-3-acetonitrile, Indole-3-carbinole (I3C), 3,3’-diindolylmethane (DIM) and Indolo(3,4)bicarbazole, which are found mainly in cruciferous vegetables like broccoli or Brussel sprouts (11). After consumption, I3C is converted in the stomach by acid-mediated condensation into various byproducts, including high affinity AhR ligands DIM and indole[3,2-b] carbazole (ICZ) (11).

Another source of nutritional AhR ligands is microbiota metabolism, particularly tryptophan catabolism. Some species of bacteria such as *Lactobacillus* can use tryptophan instead of glucose as a source of energy, and produce AhR ligands such as indole-3-acetic acid (IAA), tryptamine (TA) and 3-methyl indole (14, 15). A well-described example is *Lactobacillus reuteri*, producing the AhR ligand indole-3-aldehyde (IAld) (15). Moreover, Tryptophanase-expressing bacteria, which are mostly ampicillin-sensitive and vancomycin-resistant, degrade tryptophan into indole that is further metabolized by host liver cells into AhR ligands, such as indoxyl-3-sulfate (I3S) and indole-3-propionic acid (IPA) (16). In addition, it has been reported that butyrate and other short-chain fatty acids (SCFA), which originate from the fermentation of dietary fibers by the microbiota, can activate AhR signaling in reporter cell lines (12). Whether this observation holds true in other cell types remains to be confirmed. In particular, another study failed to detect AhR activation in B cells upon *in vitro* exposure to butyrate, while 5-hydroxyindole-3-acetic acid (5-HIAA), a serotonin metabolite, activated AhR signaling in B cells *in vitro* and *in vivo* (13).

## What Is the Biodistribution of Nutritional AhR Ligands?

Dietary AhR ligands are released locally in the digestive track but also distributed to other sites *via* the blood. After oral administration, the biodistribution of I3C and its acid condensation products has been analyzed by high-performance liquid chromatography. I3C is absorbed from the gut and distributed systemically into a number of well-perfused tissues (17). I3C level peaks at 15min and decreases considerably 1h after administration in the plasma, liver, kidney, lung, heart and brain. However, I3C products DIM, LTR_1_ and HI-IM are detectable in these organs from 15 min and persist after 6h, and up to 24h in the liver. ICZ, another product from I3C, was also identified in the liver, but not in plasma or other organs, at 6h and 24h after I3C administration (17). In line with those observations, I3C was detected in serum 15 min after gavage, but not when administered as supplement in the chow diet. By contrast, DIM was detectable in the serum of mice fed with I3C-supplemented chow diet (18). In a study carried out in women given a single oral I3C dose, DIM, but not I3C itself, was detected in plasma peaking at 2h and returning to basal levels 24h after administration (19). These observations suggest that I3C is rapidly cleared from the circulation, while its condensation products can reach distant organs and exert longer lasting effects.

It has also been proposed that nutritional AhR ligands can cross the blood-brain barrier. After intra-peritoneal injection, I3S was detected in the brain (20), however oral administration was not examined.

Finally, microbiota-derived AhR ligands have been detected in breastmilk. After gavage of pregnant mice with radiolabeled *E.Coli*, a species which expresses Tryptophanase, labelled AhR ligands were detected in maternal milk, including indole-3-lactic acid, showing the transfer from maternal intestinal microbiota to milk (21).

## Do Nutritional AhR Ligands Influence Microbiota Composition?

Tryptophan content in the diet shapes significantly the microbiota composition. Tryptophan supply relies exclusively on the diet since the host cannot synthetize it. After 2 or 3 weeks of dietary intervention, mice fed with a tryptophan-deprived diet display an increase in fecal *Actinobacteria* and *Proteobacteria*, and lower relative abundance of *Bacteroidetes* and bacteria belonging to *Firmicutes* phylum such as *Lactobacillus and Staphylococcus* (22, 23). In addition, after 4 weeks of tryptophan-low diet, the abundance of *Lactobacillus reuteri* is also decreased in the stomach (15). Of note, tryptophan is metabolized in the gut not only into AhR ligands by microbiota, but also through the serotonin and kynurenine pathways by host cells (5, 24). Whether the observed alterations in microbiota composition are entirely dependent on changes in AhR ligands availability remains to be confirmed. In addition, caution should be exercised when interpreting *in vivo* experiments employing tryptophan-low diets, as modification in microbiota diversity by itself may impact the outcome.

By contrast, I3C content in the diet only causes a relatively minor change in intestinal microbiota composition. Normal chow contains phytochemicals that can act as precursors of AhR ligands. Switching from normal chow to a synthetic diet alters fecal microbiota diversity (25, 26). Mice fed with a AhR ligand-free synthetic diet show a decreased abundance in fecal *Bacteroidetes* and increased abundance in *Actinobacteria* and *Firmicutes* compared to mice fed with the same diet supplemented with I3C (25, 26). Relative change observed in *Erysipelotrichaceae* is inconsistent between studies (25, 26). Importantly, most of these alterations in fecal microbiome composition were also observed in AhR-deficient mice, showing that this occurs independently of AhR signaling (26). In a study employing a synthetic diet supplemented with DIM, no difference were observed in fecal microbiota between groups (27). However, mice fed with the synthetic diet had increased *Bacteroidetes* and decreased *Firmicutes* abundance in the small intestine compared to mice fed with the DIM-supplemented synthetic diet. These differences in microbiota diversity induced by DIM supplementation were abrogated in AhR-deficient mice (27).

These results highlight a complex interplay between microbiota composition and the supply of dietary AhR ligands, with tryptophan having a more pronounced effect than single AhR ligands.

## What Is the Impact of Nutritional AhR Ligands on Intestinal Immunity?

Nutritional AhR ligands are essential for the maintenance of intestinal intraepithelial lymphocytes (IELs) and type 3 innate lymphoid cells (ILC3).

Small intestine IELs are a specialized population of T cells composed of several subsets (TCRγδ, CD4^-^CD8αα^+^ TCRαβ and CD4^+^CD8αα^+^ TCRαβ). Mice fed with a AhR ligand-free synthetic diet have lower numbers of TCRγδ and CD4^-^CD8αα^+^ TCRαβ IELs compared to mice on I3C-supplemented synthetic diet (28, 29). However, these IELs can develop normally in AhR-deficient, indicating that dietary AhR ligands are required for their maintenance rather than differentiation (29). In addition, CD4^+^CD8αα^+^ TCRαβ IELs are decreased in the intestine of mice fed with a tryptophan-low synthetic diet compared to standard diet and tryptophan-high diet (30). *Lactobacillus reuteri* was identified as essential for CD4^+^CD8αα^+^ TCRαβ IELs development. However, a tryptophan-high diet in conjunction with *L.reuteri* colonization was not sufficient to induce these IELs in germ-free mice, suggesting the participation of additional factors (30).

ILC3 produce lymphotoxin, controlling the development of intestinal lymphoid follicles. Mice fed with a AhR ligand-free synthetic diet display decreased ILC3 and intestinal lymphoid follicles numbers compared to mice fed on I3C-supplemented synthetic diet (28, 31). Of note, I3C supplementation had no impact when given to AhR-deficient mice (31). In addition, development of ILC3 and intestinal lymphoid follicles are normal in germ-free mice (32), suggesting that dietary AhR ligands, produced independently of microbiota metabolism, are sufficient for ILC3 differentiation or maintenance. ILC3 are the main producers of intestinal IL22, which is critical for the secretion of antimicrobial peptides by intestinal epithelial cells and the defense against intestinal infections (33). Consistent with decreased ILC3 numbers, mice fed on a AhR ligand-free synthetic diet express lower levels in the intestine of antimicrobial peptides such as C-type lectin regenerating islet-derived protein 3 (RegIII) (29, 34), and are more susceptible to infections with *Citrobacter Rodentium* (35) or *Clostridium difficile* (28).

AhR has been proposed to control Treg differentiation. Tregs in the gut express higher levels of AhR than other Tregs in the body (36). AhR expression is necessary for Treg gut homing and function, and is independent of microbiota, since it is not affected in antibiotics-treated mice or germ-free mice. Whether nutritional AhR ligands play a role is unclear, as feeding mice with an AhR ligand-free synthetic diet was reported to be inconclusive regarding Treg development (36).

Lack of dietary AhR ligands worsens the symptoms of intestinal inflammation. In the model of DSS-induced colitis, mice on a synthetic AhR ligand-free diet show more severe symptoms, such as weight loss and tissue damage, compared to mice fed with a synthetic diet supplemented with I3C (26, 29) or with tryptophan (34). Tryptophan supplementation did not modify the severity of symptoms in AhR-deficient mice, confirming the dependency on AhR activation (34). Consistent with these observations, symptoms of DSS-induced colitis were mildly ameliorated when mice fed on normal chow were given tryptophan supplementation in the drinking water (37) or I3C by oral gavage (38). In mice fed on AhR ligand-free synthetic diet, increased epithelial damage may be due to the lack of IL22 production in response to DSS-induced inflammation (26, 34). Importantly, AhR activation in intestinal epithelial cells is also involved in barrier repair during colitis (39).

Finally, in a model of oral tolerance to ovalbumin, mice fed with I3C-enriched chow diet have lower levels of serum anti-ovalbumin IgG1 antibodies, indicating better induction of oral tolerance (18). This was correlated with increased expression in the small intestine of retinaldehyde dehydrogenase, a molecule known to promote Treg differentiation, but the target cells of dietary AhR ligands in this model remain unclear.

**Figure 2:**
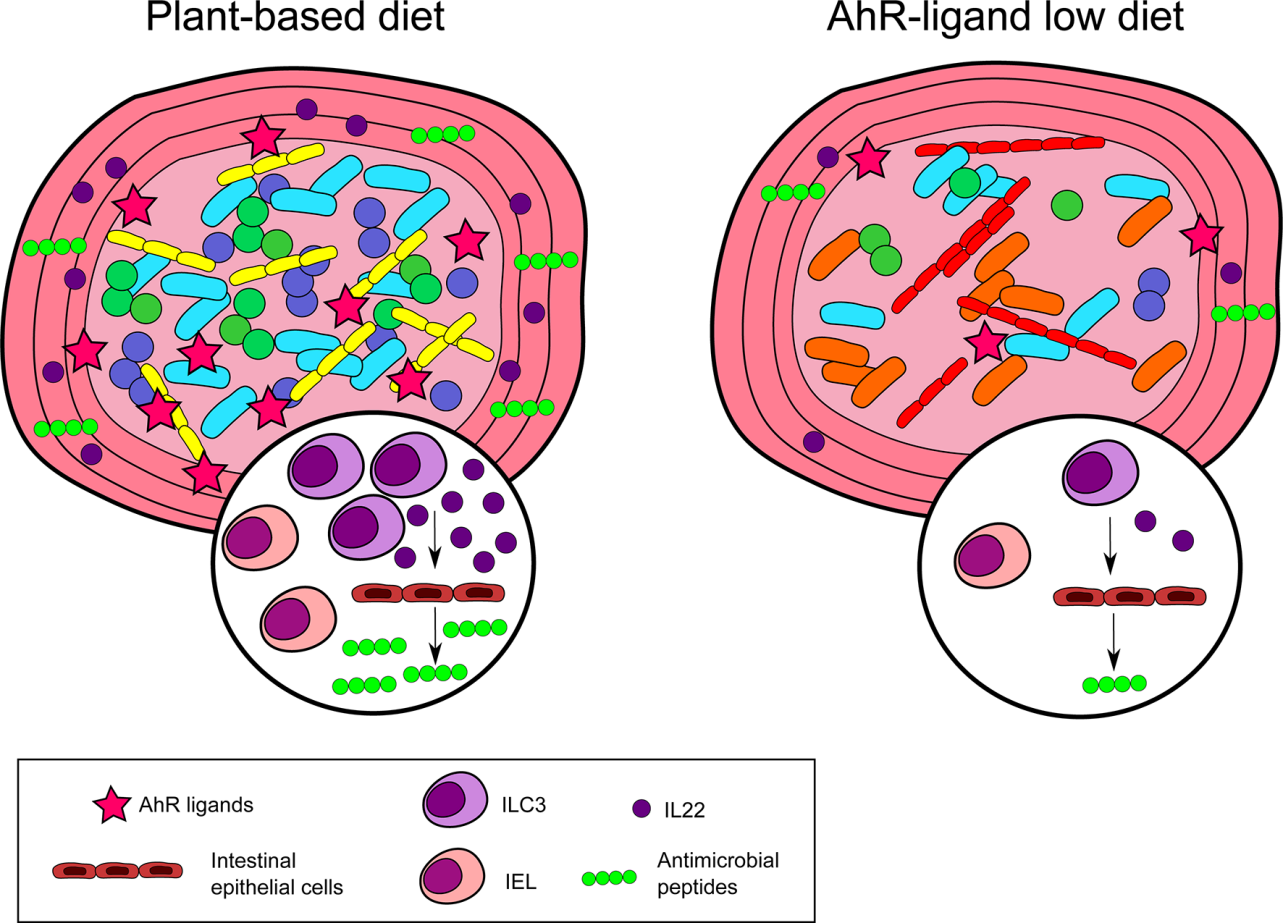
Nutritional AhR ligands in intestinal immunity. Nutritional AhR ligands are involved in the maintenance of intestinal intra-epithelial lymphocytes (IEL) and type innate-like lymphoid cells (ILC3). ILC3 are the main producers of IL22, which acts on intestinal epithelial cells to induce the secretion of antimicrobial peptides. In the absence of dietary AhR ligands, IEL and ILC3 are reduced, and microbiota diversity is altered.

Collectively, these observations show an essential role for nutritional AhR ligands in maintaining intestinal lymphoid populations and homeostasis (**Figure 2**).

## What Is the Impact of Nutritional AhR Ligands on Immune Responses at Distant Sites?

**Figure 3:**
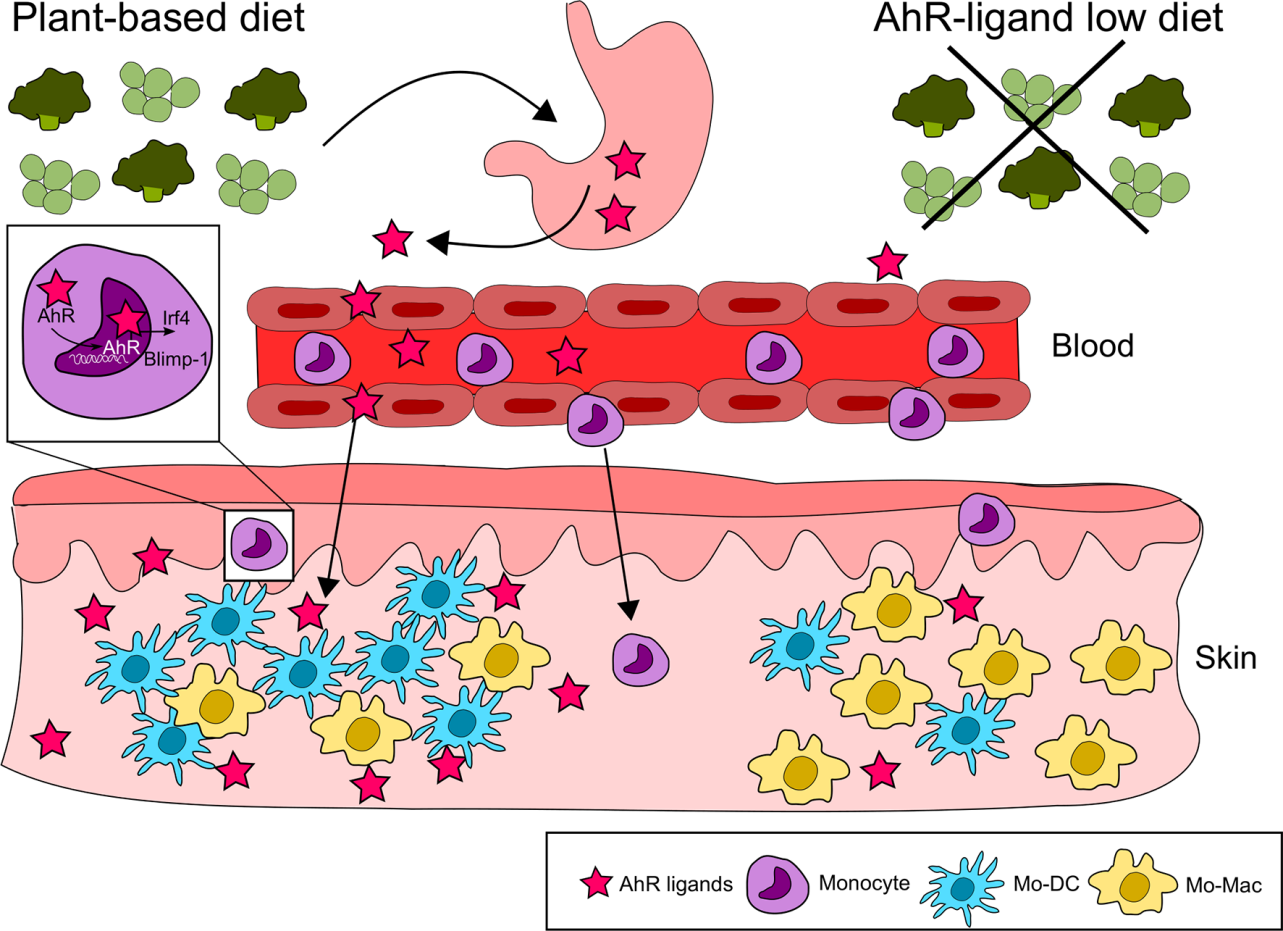
Nutritional AhR ligands in monocyte differentiation. Nutritional AhR ligands modulate monocyte differentiation in the skin. AhR activation from dietary agonists favors monocyte differentiation towards dendritic cells *via* the induction of the transcription factors Irf4 and Blimp-1. In the absence of AhR signaling, monocytes differentiate preferentially into macrophages.

Nutritional AhR ligands can also modulate the differentiation of immune cells outside of the intestinal mucosa, as shown for monocytes. Monocytes circulate in the blood and are recruited to tissues where they differentiate into dendritic cells or macrophages. In mice fed with a synthetic AhR ligand-free diet, monocyte differentiation into dendritic cells is reduced in the skin, compared to mice on an I3C-supplemented diet (40). This is consistent with *in vitro* observations that AhR activation skews monocyte differentiation from macrophages to dendritic cells by controlling the expression of the transcription factors Irf4 and Blimp-1. In addition, monocyte differentiation into dendritic cells in the peritoneum is impaired in antibiotics-treated mice and could be restored by I3C diet supplementation (40), suggesting a role for microbiota-derived AhR ligands in physiological conditions (**Figure 3**).

Deficit in dietary AhR ligands increases the severity of inflammation in the central nervous system, as evidenced in the experimental autoimmune encephalomyelitis (EAE) model. After induction of EAE, mice placed on a synthetic tryptophan-free diet display worse disease scores and delayed recovery compared to mice fed with tryptophan-supplemented diet (20, 41). This difference was abolished in mice deficient for AhR specifically in astrocytes or in microglia, showing that dietary AhR ligands can exert their effect on brain-resident populations. Lack of dietary tryptophan results in the increased expression of pro-inflammatory molecules in the brain such as *Ccl2, Nos2* and *Tnfa* (20, 41). Treatment with ampicillin also delays disease recovery and increases *Ccl2* and *Nos2* expression, which could be reverted by diet supplementation with IPA, IAld, indole or Tryptophanase (20), suggesting a major role in this phenomenon for microbiota-derived AhR ligands.

AhR ligands from microbiota metabolism also influence the differentiation of IL-10 producing regulatory B cells, which are found in lymphoid organs. Gavage with 5-HIAA increases the expression of *Il10* in spleen B cells and reduces the severity of joint swelling in a model of antigen-induced arthritis (13). This effect is abolished in mice deficient for AhR in B cells, confirming the role of AhR signaling.

These observations indicate that AhR ligands participate in the communication between gut, microbiota and distant tissues such as brain and skin.

## Conclusion and Perspectives

There is accumulating evidence that nutritional AhR ligands play an essential role in the maintenance of intestinal immune homeostasis and the control of intestinal inflammation. Several studies also suggest a similar role in distant tissues such as skin and brain. Whether dietary AhR ligands impact other organs or mucosal sites remains to be investigated.

Circadian rhythms regulate some essential aspects of immune activity, such as leukocyte trafficking or inflammatory cytokine secretion (42). AhR has been reported to interact with circadian clock proteins and suppress their transcriptional activity (43). In addition, the supply of nutritional AhR ligands fluctuates across time with feeding behavior, as does microbiota mass and the release of nutritional metabolites (44). It will be important to decipher the possible connection between AhR, circadian rhythms and immune cells.

Impaired production of AhR ligands has been observed in the intestinal microbiota of patients suffering from inflammatory bowel disease (45) and celiac disease (22). Given the critical impact of dietary AhR ligands on ILC3 numbers and the maintenance of barrier integrity, diet supplementation with AhR ligands, or AhR ligand-producing bacteria, is an attractive strategy to improve the treatment of inflammatory gastrointestinal diseases (24). However, a better understanding of the role of nutritional AhR ligands on the immune homeostasis of distant tissues and on myeloid cells will be essential to optimize these therapeutic approaches.

## Author Contributions

AJ and ES wrote the manuscript. All authors contributed to the article and approved the submitted version.

## Funding

This work was funded by INSERM, Institut Curie, Agence Nationale de la Recherche (ANR-10-LABX-0043 and ANR-17-CE15-0011-01) and European Union’s Horizon 2020 research and innovation programme under the Marie Sklodowska-Curie grant agreement No 842535.

## Conflict of Interest

The authors declare that the research was conducted in the absence of any commercial or financial relationships that could be construed as a potential conflict of interest.
